# Intestinal fistula accompanied by recurrent peritonitis associated with peritoneal dialysis: a case report

**DOI:** 10.1186/s12876-020-01303-1

**Published:** 2020-05-24

**Authors:** Qiuyuan Shao, Yangyang Xia, Qingyan Zhang, Miao Zhang

**Affiliations:** Department of Nephrology, Nanjing Drum Tower Hospital, The Affiliated Hospital of Nanjing University Medical School, Nanjing, Jiangsu Province, 210008 China

**Keywords:** Peritoneal dialysis, Intestinal fistula, Computed tomography, Electronic colonoscopy, Chronic kidney disease

## Abstract

**Background:**

Intestinal perforation from peritoneal dialysis is rare, but the resulting complications are serious. Some patients do not necessarily have symptoms, and it can be difficult to differentiate their condition from PD-related (peritoneal dialysis-related) peritonitis, which may lead to misdiagnosis. Here we report a peritoneal dialysis patient with intestinal fistula associated with recurrent peritonitis.

**Case presentation:**

A 44-year-old man had been treated for more than 6 years with peritoneal dialysis for chronic kidney disease stage-V. Abdominal computed tomography and electronic colonoscopy revealed an appendiceal fossa with adjacent fistula. The peritoneal dialysis catheter was removed, and the patient recovered with no recurrence of complications.

**Conclusion:**

We report a case of a rare complication of peritoneal dialysis. The intestinal fistula in this patient was mainly caused by recurrent peritonitis and removal of the catheter could control the peritonitis.

## Background

Owing to its safety and effectiveness, peritoneal dialysis is widely used in patients when acute or chronic renal failure reaches end-stage renal disease (ESRD). Some complications, such as peritonitis, pain, flow restriction, and exit-site leak, are common [1]. According to the literature, intestinal fistula is a rare complication of peritoneal dialysis [2]. The symptoms of intestinal perforation include watery diarrhea, difficulty in draining, and peritonitis [3]. If treatment is delayed, the consequences can be serious. Here, we report a case of intestinal fistula caused by peritoneal dialysis.

## Case presentation

A 44-year-old man had been treated with peritoneal dialysis for chronic kidney disease stage-V (CKD-V) for more than 6 years. The ESRD was caused by chronic nephritis. He has a history of hypertension for 6 years without other particular disease. He stated had edema of eyelid and low limbs intermittently and weakness of whole body. A peritoneal dialysis catheter (a right side straight two cuffed Tenckhoff catheter) was placed in August 2012, and the patient received peritoneal dialysis regularly since that time. Several episodes of peritonitis caused by peritoneal dialysis lasted more than 4 weeks only with symptomatic and empirical treatment. In May 2019, Leakage of peritoneal dialysis fluid was noted at the exit-site of the PD catheter. Three days later, the patient developed chills and fever and diagnosed with peritonitis (Supplementary Table 1). After a week of antibiotic therapy, (Imipenem 0.5 g Intraperitoneal for 4 h once, Meropenem 0.5 g Intravenous infusion Q12h), his body temperature returned to normal; however, the peritoneal dialysis effluent became turbid, and passage of watery stool occurred immediately after each infusion of peritoneal dialysate into the abdominal cavity. The patient had no abdominal pain or distention.

**Fig. 1:**
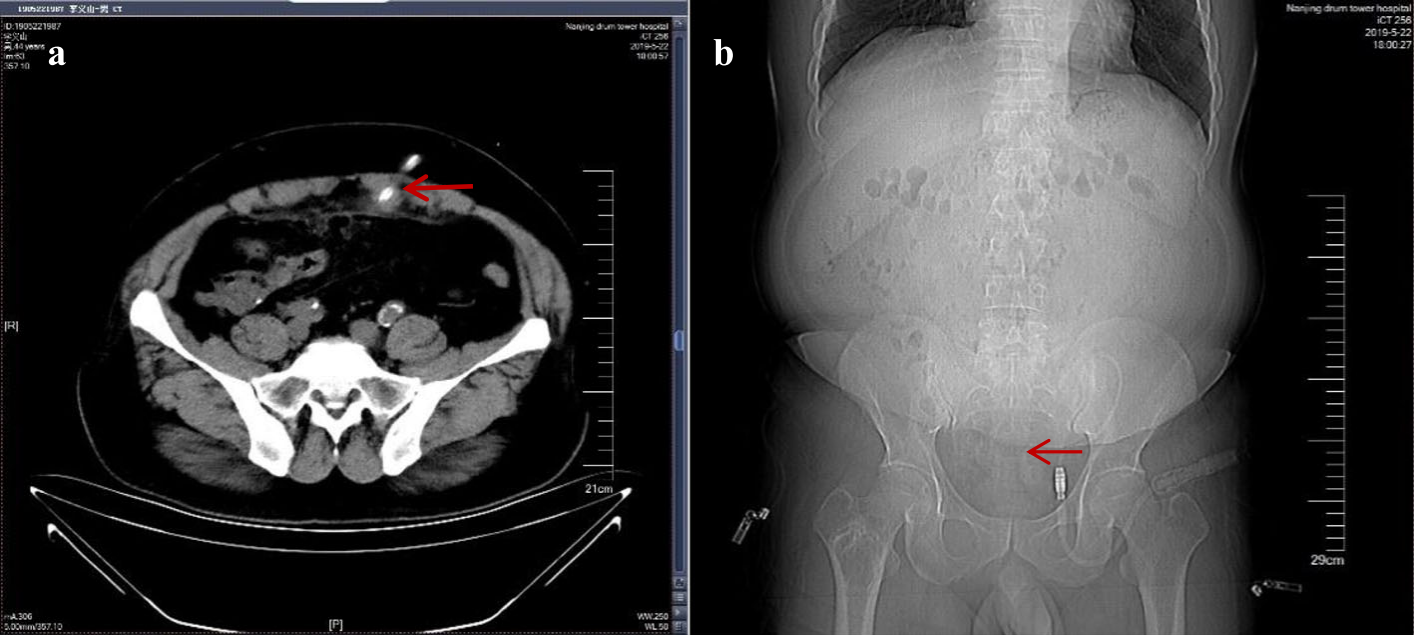
**a, b** Abdominal computed tomography and radiography showing proper catheter alignment

**Fig. 2:**
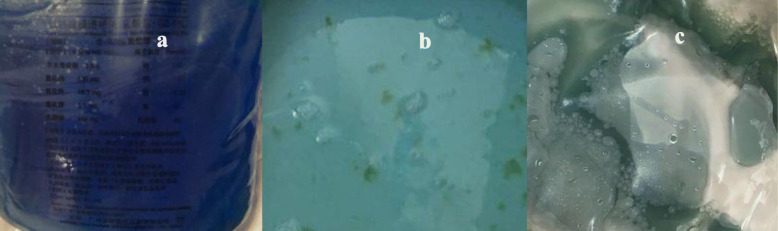
Contrast chart of peritoneal dialysate and methylene blue. **a**. Peritoneal dialysate containing methylene blue reagent. **b**. Pale blue anal effusion. **c**. Light blue drainage of peritoneal dialysate

**Fig. 3:**
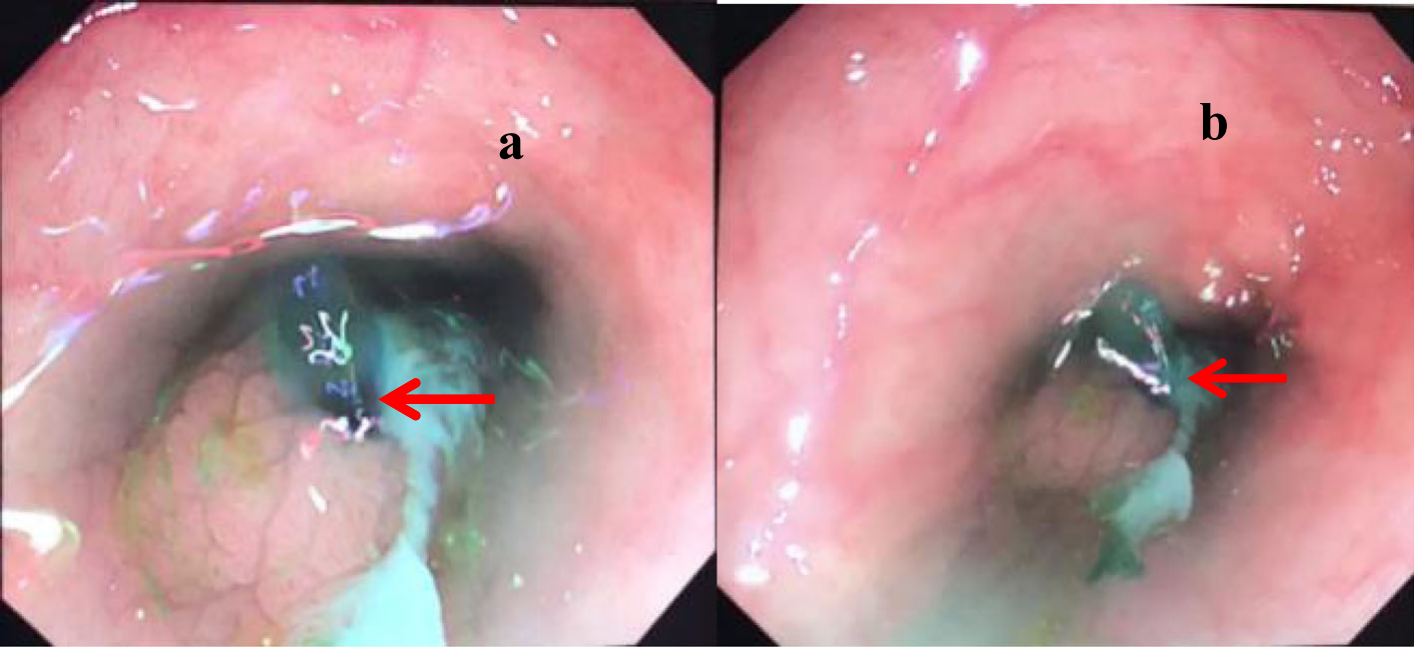
Colonoscopy revealing the colonic lumen after injection of methylene blue-containing dialysate into the abdominal lumen. The picture was obtained near the appendiceal orifice

Abdominal computed tomography (CT) revealed inflammation in the abdominal cavity, extensive peritoneal calcification, and appropriate positioning of the peritoneal catheter, but intestinal perforation was not evident (Fig. 1). Peritoneal dialysate containing methylene blue reagent was injected into the abdominal cavity. After 2 hours, anal drainage was light blue (Fig. 2). Thus, an intestinal fistula was suspected. Colonoscopy revealed methylene blue at the area of the appendiceal orifice (Fig. 3), which confirmed the presence of a communication between the abdominal cavity and the bowel lumen. Peritoneal dialysis was discontinued and hemodialysis was initiated. The peritoneal catheter was removed by open surgery. No abdominal pain, abdominal distension, or other symptoms occurred during the follow-up period, which lasted a minimum of 3 months.

## Discussion and conclusion

### Discussion

Peritoneal dialysis is widely used in patients with chronic renal failure because of its safety and effectiveness. However, complications, such as peritonitis, abdominal pain, and intestinal fistula, may occur [1, 2]. Even with strict operating standards, complications cannot be avoided. The diagnostic features of intestinal perforation in peritoneal dialysis patients include watery diarrhea, difficulty in drainage of the peritoneal dialysis fluid, and symptoms and signs of peritonitis [3]. If treatment is delayed, the consequences can be serious; the reported mortality rate is as high as 46–57% [4]. Once an intestinal fistula occurs, it must be treated as soon as possible, and the peritoneal dialysis catheter should be removed promptly. Surgical repair of the fistula may be needed. Most dialysis-associated perforations occur in the colon, followed by the cecum and rectum [1, 2, 4–6], whereas perforation of the small intestine is rare.

Intestinal perforation associated with peritoneal dialysis can be divided into acute and chronic forms. Acute perforation may occur with implantation of the dialysis catheter. Extensive peritoneal calcification, which may develop after repeated peritonitis, can predispose to bowel perforation [7]. In our patient, we suspect that repeated peritoneal dialysis-related peritonitis led to the intestinal perforation, which has rarely been reported. Other factors include intestinal tumors, mesenteric ischemic diseases, which may also cause intestinal perforation [8]. Patients with recurrent abdominal infections are prone to bacterial or fungal peritonitis and are also at high risk for intestinal perforation. Enlargement of the kidneys caused by polycystic kidney disease may lead to increased intra-abdominal pressure and intestinal perforation [9]. Perforation of an inflamed intestinal diverticulum is regarded as a major cause of intestinal perforation. The reported incidence of colonic diverticula in patients with end-stage polycystic kidney disease is high, at approximately 80% [10].

In addition to intestinal perforation from catheter implantation, catheters can play a role in perforation due to factors such as improper position of the catheter, retention of the catheter for a long time after the cessation of peritoneal dialysis, and repeated rubbing of the catheter against the bowel wall [5]. Catheter insertion methods include percutaneous procedure with or without image guidance, open surgical dissection, peritoneoscopic procedure, and surgical laparoscopy [11]. Brown et al. [12] reported that in a series of 435 patients who had catheters implanted by using these techniques, the Moncrief and Popovich method alone may not induce intestinal perforation. Rubin et al. [13] reported an incidence of perforation of 0.1% using the Moncrief and Popovich technique. Fujiwara et al. [6] emphasized that catheter-related intestinal perforation can be due to the presence of unused catheters, typically 1.6–48 months after the use has ceased. It is proposed that long duration of an immobile catheter in the peritoneal cavity containing little fluid may cause pressure necrosis of the bowel. Thus, if peritoneal dialysis is no longer being performed, the catheter should be removed or flushed regularly. Unidirectional intestinal fistula present after removal of the catheter is safe because if the intestinal fistula is not open, diffuse peritonitis will not occur.

It is often difficult to determine whether clinically suspected peritonitis in patients on peritoneal dialysis is due to dialysis [14]. Moreover, intestinal perforation in such patients may be unrecognized because of the lack of symptoms [15, 16]. Therefore, it is important to use imaging and endoscopic methods for detecting intestinal perforation in these patients.

### Conclusion

A case of intestinal perforation associated with long-term peritoneal dialysis and repeated episodes of inadequately treated peritonitis is presented. This associated complication of peritoneal dialysis may be difficult to diagnose; however, it should be suspected, and when present, it should be treated promptly.

## Supplementary information

**Additional file 1: Table S1.** Routine analysis of ascitic fluid

## Data Availability

The datasets used and/or analysed during the current study available from the corresponding author on reasonable request.

## Abbreviations

CKD: chronic kidney disease
CT: computed tomography
