# Supplementary material for: Intestinal fistula accompanied by recurrent peritonitis associated with peritoneal dialysis: a case report
Source: BMC Gastroenterol. 2020 May 24;20:157. doi: 10.1186/s12876-020-01303-1 (PMC7245831; doi:10.1186/s12876-020-01303-1)
Supplement: Supplementary file 1 — Additional file 1: Table S1. Routine analysis of ascitic fluid [file 12876_2020_1303_MOESM1_ESM.docx]

**Supplementary**

**Table 1 Routine analysis of ascitic fluid**

| Parameters | Results |
| --- | --- |
| polymorphonuclear (PMN) cell count | 43384.0×10^6/L↑ |
| Protein | Positive ↑ |
| Lymphocyte ratio | 1% |
| Neutrophil ratio | 95% |
| Monocyte ratio | 4% |
| Lymphocyte count | 434×10^6/L |
| Neutrophil count | 41215×10^6/L |
| Monocyte count | 1735×10^6/L |
| Ascitic fluid culture | Escherichia coli positive ↑ |
| Drug sensitivity | Cefoperazone Sodium / Sulbactam, Piperacillin / Tazobactam, Ceftetam, Ertapenem, Imipenem, Amikacin, Gentamicin, Tobramycin |
